# C-Reactive Protein, Advanced Glycation End Products, and Their Receptor in Type 2 Diabetic, Elderly Patients with Mild Cognitive Impairment

**DOI:** 10.3389/fnagi.2015.00209

**Published:** 2015-10-29

**Authors:** Malgorzata Gorska-Ciebiada, Malgorzata Saryusz-Wolska, Anna Borkowska, Maciej Ciebiada, Jerzy Loba

**Affiliations:** ^1^Department of Internal Medicine and Diabetology, Medical University of Lodz, Lodz, Poland; ^2^Department of General and Oncological Pneumology, Medical University of Lodz, Lodz, Poland

**Keywords:** AGEs, cognitive impairment, diabetes, elderly, RAGE

## Abstract

**Objective:**

The aim of the study was to evaluate serum levels of advanced glycation end products (AGEs), receptor for advanced glycation end products (RAGE), and C-reactive protein (CRP) in elderly patients with type 2 diabetes mellitus with and without mild cognitive impairment (MCI) and to determine the predictors (including AGEs, RAGE, and CRP levels) of having MCI in elderly patients with type 2 diabetes.

**Methods:**

Two hundred seventy-six diabetics elders were screened for MCI (using the Montreal Cognitive Assessment: MoCA score). Data of biochemical parameters and biomarkers were collected.

**Results:**

Serum AGEs, RAGE, and CRP levels were significantly increased in MCI patients compared to controls. In group of patients with MCI, serum RAGE level was positively correlated with AGEs level and with CRP level. RAGE, AGEs, and CRP concentrations were positively correlated with HbA1c levels and negatively correlated with MoCA score. The univariate logistic regression models revealed that variables, which increased the likelihood of diagnosis of MCI in elderly patients with type 2 diabetes were higher levels of HbA1c, RAGE, AGEs, CRP, TG, lower level of HDL cholesterol, previous CVD, HA, or use of HA drugs, hyperlipidemia, retinopathy, nephropathy, increased number of co-morbidities, older age, and less years of formal education. HA or use of HA drugs, previous CVD, higher level of RAGE and CRP, older age and less years of formal education are the factors increasing the likelihood of having MCI in elderly patients with type 2 diabetes in multivariable model.

**Conclusion:**

In summary, serum AGEs, RAGE, and CRP are increased in the circulation of MCI elderly diabetic patients compared to controls. A larger population-based prospective study needs to be performed to further confirm the relationship between AGEs, RAGE, and the cognitive decline or progress to dementia.

## Introduction

Diabetes mellitus is one of the important chronic diseases worldwide.

The prevalence of type 2 diabetes mellitus (T2DM) raised rapidly in the last few decades (Wild et al., 2004). Complications include small and large vessels, eyes, kidneys, cranial, and peripheral nerves. Recent studies suggested that diabetes is a risk factor for cognitive decline and dementia in the elderly (Cukierman et al., 2005; Gorska-Ciebiada et al., 2009, 2014). T2DM has a highly complicated pathogenesis, so the etiology of cognitive impairment in diabetes is probably associated with many factors (Strachan et al., 1997). One of the recent hypotheses suggests that low-grade chronic inflammation is associated with mild cognitive impairment (MCI) or with cognitive decline (Yaffe et al., 2003; Schuitemaker et al., 2008). MCI is a transitional stage between normal cognitive aging and dementia. One large study has proven that some inflammatory parameters, such as C-reactive protein, TNF-α, and IL-6, are connected with cognitive dysfunction and decline (Yaffe et al., 2003). There are evidences from other population-based study that there are significant associations between CRP and MCI and with the attention and executive function domain score (Roberts et al., 2009). Another potential marker of cognitive dysfunction is a receptor for advanced glycation end products (RAGE). RAGE is a transmembrane receptor that belongs to the immunoglobulin super family of cell surface receptors. It interacts with its ligands and causes persistent inflammatory responses at vascular wall and vascular injuries, a major complication of diabetes (Neeper et al., 1992; Hori et al., 1996; Schmidt et al., 2000a; Kumano-Kuramochi et al., 2009). The major RAGE ligands in diabetes are advanced glycation end products (AGEs). They are derivatives of proteins, lipids, and ribonucleic acids. Serum AGEs levels increases with age and with diabetes or hyperglycemic states (Ulrich and Cerami, 2001). RAGE-mediated mechanisms play a crucial role in diabetes complications because they induce reactive oxygen species-mediated inflammation in vascular wall (Win et al., 2012). RAGE is also an important cell-signaling receptor involved in cognitive impairment. Recent studies report the association between RAGE levels and cognitive impairment in Alzheimer’s disease (Deane and Zlokovic, 2007). Increased expression of RAGE was found in neurons and microglia in the hippocampus (Lue et al., 2001). Some authors have suggested that RAGE and other receptors could be involved in the injury of the brain in Alzheimer’s disease and diabetes by microglial activation and oxygen-mediated neuroinflammation (Lue et al., 2012).

Data concerning serum AGEs and RAGE levels in MCI subjects with diabetes are lacking; therefore, the aim of the study was to (1) evaluate serum levels of AGEs, RAGE, and CRP in elderly patients with T2DM with and without MCI and (2) determine the predictors (including AGEs, RAGE, and CRP levels) of having MCI in elderly patients with T2DM.

## Materials and Methods

### Study Population

A survey was conducted among unselected 276 elders who attended to outpatient clinic belonged to the Department of Internal Medicine and Diabetology, University Hospital no 1 in Lodz, Poland. A brief screening for recruitment was conducted by the investigators to identify potential participants. We included patients aged 65 and over with diabetes type 2 diagnosed minimum 1 year earlier, subjects who had been able to understand and cooperate with study procedures. The exclusion criteria were diagnosed depression or dementia, use of possible or known cognition-impairing drugs in the previous 3-month, presence of neoplasm, constant alcohol or substance abuse, severe visual, mobility, or motor coordination impairment, history of head trauma, inflammatory or infectious brain disease, severe neurological or psychiatric illness (Table 1).

**Table 1:** **The inclusion and exclusion criteria of the study**.

**The inclusion criteria**
Age ≥65 years
Diabetes type 2 diagnosed minimum 1 year earlier
Full ability to understand and cooperate with study procedures
**The exclusion criteria**
Diagnosed depression or dementia
Use of possible or known cognition-impairing drugs in the previous 3-month
Presence of neoplasm
Constant alcohol or substance abuse
Severe visual, mobility, or motor coordination impairment
History of head trauma
Inflammatory or infectious brain disease
Severe neurological or psychiatric illness

Written informed consent was obtained from the participants at the beginning of the study. The first part of visit included a morning blood draw after a 10- to 12-h overnight fast, blood pressure measurements, height and weight assessment, and complete physical examination. Then, patients had eaten a breakfast followed by capillary glucose level measuring to ensure that participants were not hypoglycemic at the time of cognitive testing. The second part of visit took place in a private area in the clinic. Subjects completed a questionnaire describing baseline demographics and underwent cognitive testing.

### Participant Characteristics, Clinical Evaluation, and Risk Factor Assessment

Demographic variables and possible risk factors were recorded in a standardized interview. Weight and height were measured to calculate body mass index [BMI = weight/height^2^ (kg/m^2^)]. The systolic and diastolic blood pressures (millimeter of mercury) were measured with the patient in sitting position after 5 min of rest. The detailed medical history of diabetes type 2 was taken and includes diabetes duration, currant treatment for diabetes and complications if present, family history of diabetes, co-morbid diseases of the patient [hyperlipidemia, hypertension, cardiovascular disease (CVD), lung disease, cancer, gastrointestinal tract diseases] and their treatment. Educational level was recorded in years of education. Smoking was classified as current, past, or never. Physical activity was recorded if any present. Diabetic vascular complications were assessed based on the existence of nephropathy, retinopathy, neuropathy, CVD, and stroke. Hypertension was defined as either a history of hypertension or use of any antihypertensive agents, Hyperlipidemia defined as use of any lipid lowering agent or an untreated serum LDL cholesterol level 2.6 mmol/l or/and triglycerides 1.7 mmol/l.

### Blood Biochemistry

After overnight fasting, blood samples were taken by venipuncture to assess serum levels of glycosylated hemoglobin (HbA1c), total cholesterol, triglycerides, low-density lipoprotein cholesterol (LDL-C), and high-density lipoprotein cholesterol (HDL-C). All the parameters were measured in a centralized laboratory.

### Determination of Serum AGEs, RAGE, and CRP

The serum levels of AGEs, RAGE were assessed using ELISA kit (EIAab, Wuhan, China), and CRP was determined by Quantikine Human Immunoassay ELISA kit (R&D System, Minneapolis, MN, USA) according to the instructions of the manufacturer. Minimum detectable concentrations were 0.78 ng/ml for AGEs and 4.12 pg/ml for RAGE, 1 × 10^−5^ mg/l for CRP.

### Neuropsychological Evaluations

All participants underwent the following tests: the Montreal Cognitive Assessment (MoCA) (Nasreddine et al., 2005) to evaluate the cognitive impairment, long version of the geriatric depression scale (GDS-30) (Yesavage et al., 1982–1983) to assessed the depressive mood, Katz Basic Activities of Daily living (BADL) and Lawton instrumental activities of daily living (IADL) questionnaires to collect information on daily activities (Lawton and Brody, 1969; Katz et al., 1970). The MoCA tests 8 cognitive domains, visual–spatial ability, attention, executive function, immediate memory, delayed memory, language, abstraction, calculation, and orientation, for a maximum total score of 30. The normal MoCA score is ≥26, with one point added if the subject has fewer than 12 years of formal education. The MoCA is better than other tools to detect MCI in the elderly patients with type 2 diabetes (Alagiakrishnan et al., 2013). MCI was diagnosed based on criteria established by the 2006 European Alzheimer’s Disease Consortium, which are currently available standard test (Petersen, 2004; Portet et al., 2006). These criteria included absence of dementia. The cut-off points for MoCA scores (19/30) are recommended for the diagnosis of “dementia” in epidemiological studies. Patients with score 19 and below were excluded from the study as dementia and sent to psychiatrist for further care. The criteria mentioned above included also absence of major repercussions on daily life (in our study, measured by Katz BADL and Lawton IADL).

This interview was followed by GDS for mood assessment (Yesavage et al., 1982–1983). GDS consists of 30 items. Scores ranging from 0 to 9 were considered as normal, and 10 to 19 were considered to have depressive symptoms. Score 20 and above was excluded from the study as severe depressive symptomatology and sent to psychiatrist for further diagnosis.

According to criteria mentioned above 276 older subjects with diabetes type 2 were selected into groups: patients with MCI and controls (patients without MCI).

### Ethics

The study was operated in accordance with the World Medical Association’s Declaration of Helsinki. Each participant was assigned a number by which he/she was identified to keep his or her privacy. The approval was obtained from the independent local ethics committee of Medical University of Lodz No RNN/420/13/KB. The purpose, nature, and potential risks of the experiments were fully explained to the subjects, and all subjects gave written informed consent at the beginning of the study. The subjects had the full capacity to consent because they maintained general cognitive function and daily activities. We included only patients who had been fully able to understand and cooperate with study procedures. We excluded subjects with diagnosed depression or dementia. There was not any surrogate consent procedure (e.g., whereby next of kin or legally authorized representative) consented on the behalf of participants.

### Statistical Analysis

All data are presented as means ± SD. Normality of distributions was assessed using the Shapiro–Wilk tests. The descriptive statistics the continuous variables using the Student’s *t*-test or the Mann–Whitney *U* test whenever applicable and non-continuous variables using χ^2^ test. Pearson correlation analysis for normally distributed variables and Spearman rank correlation for non-normally distributed variables were used to assess relationships. Simple logistic regression model was done in order to select the so-called independent factors that increase the selection risk of MCI in elderly patients with type 2 diabetes. Then, multivariable regression model was done in order to select the “strongest” factor from independent risk factors. To “optimize” the multivariable model, a stepwise approach was used (backward elimination with Wald criteria). Odds ratios (OR) were computed and presented with the 95% interval of confidence (CI). A *p*-value of <0.05 was considered statistically significant. Statistica 10.0 (StatSoft, Poland, Krakow) was used for analysis.

## Results

### General Description of MCI Subjects and Controls

Table 2 describes the baseline characteristics of the study group. Compared with controls patients with MCI were significantly older, less educated, had a longer duration of diabetes, more were diagnosed with CVD, hypertension, hyperlipidemia, retinopathy, nephropathy, and other co-morbidities. MoCA score was significantly lower in subjects with cognitive impairment. The mean level of HbA1c and triglycerides was significantly higher, and level of HDL was lower in patients with MCI compared to controls. Furthermore, CRP was found to be increased in MCI patients compared to control group (*p* < 0.001). Lastly, there were no significant differences between the groups in sex, BMI, history of smoking, stroke, presence of neuropathy, depressive syndrome, type of the treatment, systolic and diastolic blood pressure, the plasma levels of fasting glucose, and total and LDL cholesterol (*p* > 0.05).

**Table 2:** **Demographic and clinical characteristics of type 2 diabetic elderly patients**.

	All subjects	MCI	Controls	χ^2^/*Z*	*p*-value
Number of patients	276	87	189		
Age (years)*	73.6 ± 4.8	75.7 ± 4.6	72.6 ± 4.6	−4.96	<0.001
Gender (female/male)	149/127	53/34	96/93	2.46	0.12
Education-years*	11.3 ± 2.4	9.7 ± 1.8	12.0 ± 2.2	7.97	<0.001
Smoked tobacco regularly	93 (33.7%)	26 (29.8%)	67 (35.4%)	0.83	0.36
Duration of T2DM (years)*	8.69 ± 6.23	11.25 ± 6.3	7.51 ± 5.85	−5.96	<0.001
Microvascular complications Retinopathy (%)*	121 (43.8%)	61 (70.1%)	60 (31.7%)	35.6	<0.001
Nephropathy (%)*	97 (35.1%)	43 (49.4%)	54 (28.5%)	11.37	0.007
Neuropathy (%)	56 (20.2%)	20 (22.9%)	36 (19.04%)	0.57	0.45
Macrovascular complications Previous CVD (%)*	109 (39.5%)	71 (81.6%)	38 (20.1%)	94.3	<0.001
Stroke (%)	14 (5.07%)	7 (8.04%)	7 (3.7%)	2.33	0.13
Previous HA/use of HA drugs (%)*	213 (77.2%)	80 (91.95%)	138 (73.01%)	18.3	<0.001
Hyperlipidemia (%)*	218 (78.9%)	81 (93.1%)	132 (69.8%)	12.87	<0.001
Co-morbidity (*n*)*	4.66 ± 3.11	7.07 ± 3.22	3.55 ± 2.33	−8.15	<0.001
Depressive syndrome (%)	82 (29.7%)	25 (28.7%)	57 (30.2%)	0.06	0.81
Treatment Insulin (%)	130 (47.1%)	42 (48.2%)	88 (46.5%)	0.07	0.79
OAD (%)	222 (80.4%)	71 (81.6%)	151 (79.8%)	0.11	0.74
MoCA score*	25.6 ± 3.07	21.6 ± 1.5	27.4 ± 1.3	13.34	<0.001
BMI (kg/m^2^)	29.9 ± 3.67	30.4 ± 3.59	29.6 ± 3.68	−1.92	0.054
Systolic blood pressure (mmHg)	136.2 ± 15.9	136.5 ± 16.4	136.05 ± 15.8	−0.25	0.79
Diastolic blood pressure (mmHg)	75 ± 7.9	75.1 ± 8.0	74.9 ± 7.8	−0.09	0.92
Fasting plasma glucose (mmol/l)	129.3 ± 26.1	129.8 ± 27.2	129.1 ± 25.6	−0.14	0.88
HbA1c (%)*	7.24 ± 0.68	7.73 ± 0.71	7.01 ± 0.54	−7.5	<0.001
Serum cholesterol (mmol/l)	10.3 ± 2.18	10.31 ± 2.2	10.29 ± 1.71	−0.5	0.61
Serum LDL-C (mmol/l)	6.06 ± 1.67	6.01 ± 1.64	6.08 ± 1.73	−0.2	0.86
Serum triglycerides (mmol/l)*	9.65 ± 2.23	10.59 ± 2.68	9.22 ± 1.84	−6.6	<0.001
Serum HDL-C (mmol/l)*	2.5 ± 0.51	2.3 ± 0.6	2.67 ± 0.42	6.34	<0.001
CRP (mg/L)*	5.08 ± 2.8	7.6 ± 2.7	3.9 ± 2.0	−9.79	<0.001
AGEs (ng/ml)*	1.4 ± 1.06	2.19 ± 1.12	1.04 ± 0.82	−8.1	<0.001
RAGE (ng/ml)*	2.67 ± 1.68	4.24 ± 1.89	1.94 ± 0.91	−9.7	<0.001

*Significance, p < 0.05; comparing patients with MCI and those without MCI (controls).

*T2DM, diabetes type 2; OAD, oral anti-diabetic drug; CVD, cardiovascular disease; HA, hypertension; BMI, body mass index; CHOL, total cholesterol; CRP, C-reactive protein*;

*AGEs, advanced glycation end products; RAGE, receptor for advanced glycation end products; HbA1c, glycosylated hemoglobin; HDL-C, high-density lipoprotein cholesterol; LDL-C, low-density lipoprotein cholesterol; MoCA, Montreal Cognitive Assessment*,

*Data are mean − SD values. Mann–Whitney U test (Z), or χ^2^ test was used to test for significant differences*.

### Serum RAGE, AGEs, and CRP in MCI and controls

**Figure 1:**
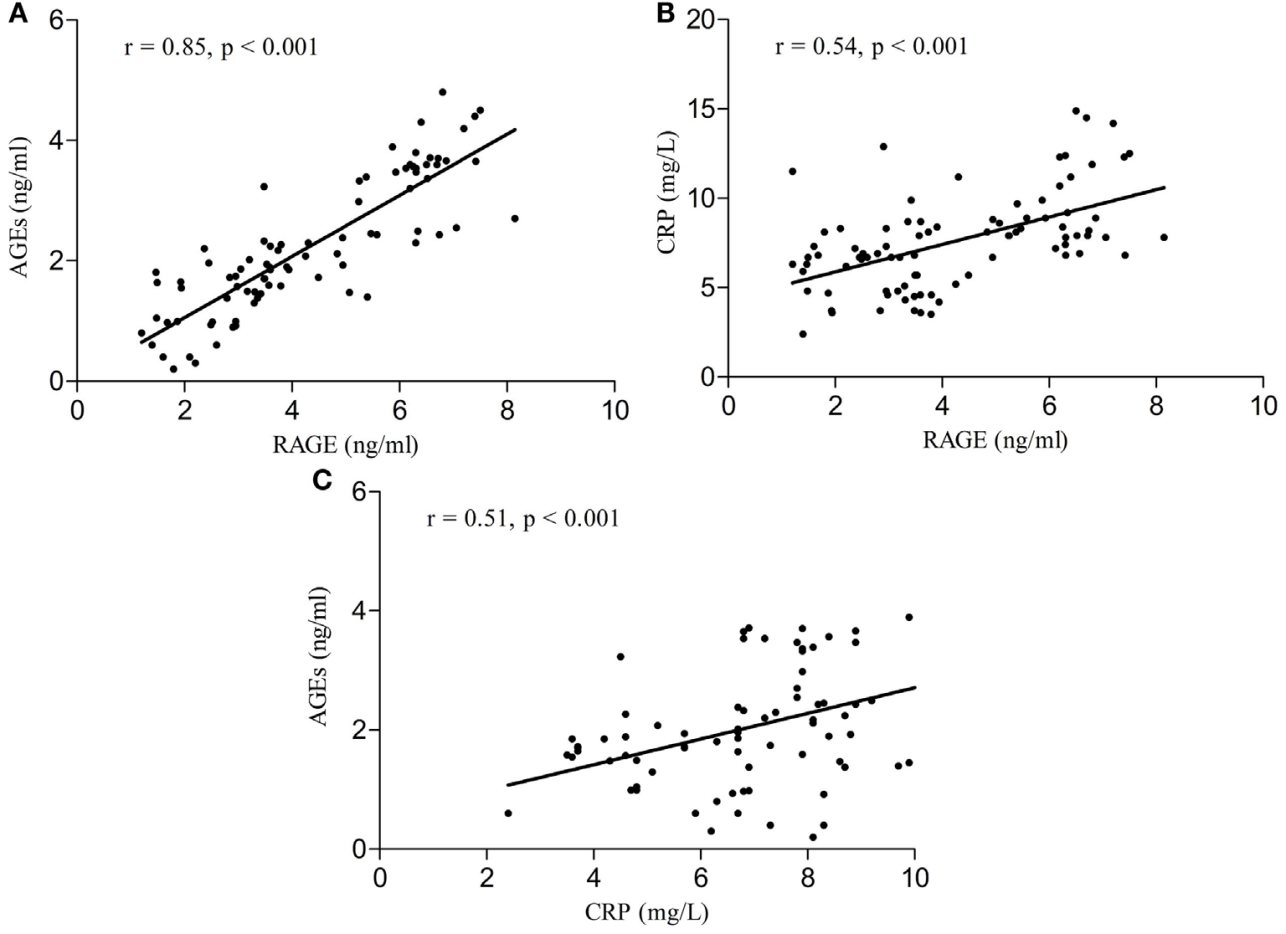
**(A)** Correlation of RAGE with AGEs in group of diabetic elderly patients with MCI. **(B)** Correlation of RAGE with CRP in group of diabetic elderly patients with MCI. **(C)** Correlation of AGEs with CRP in group of diabetic elderly patients with MCI.

**Figure 2:**
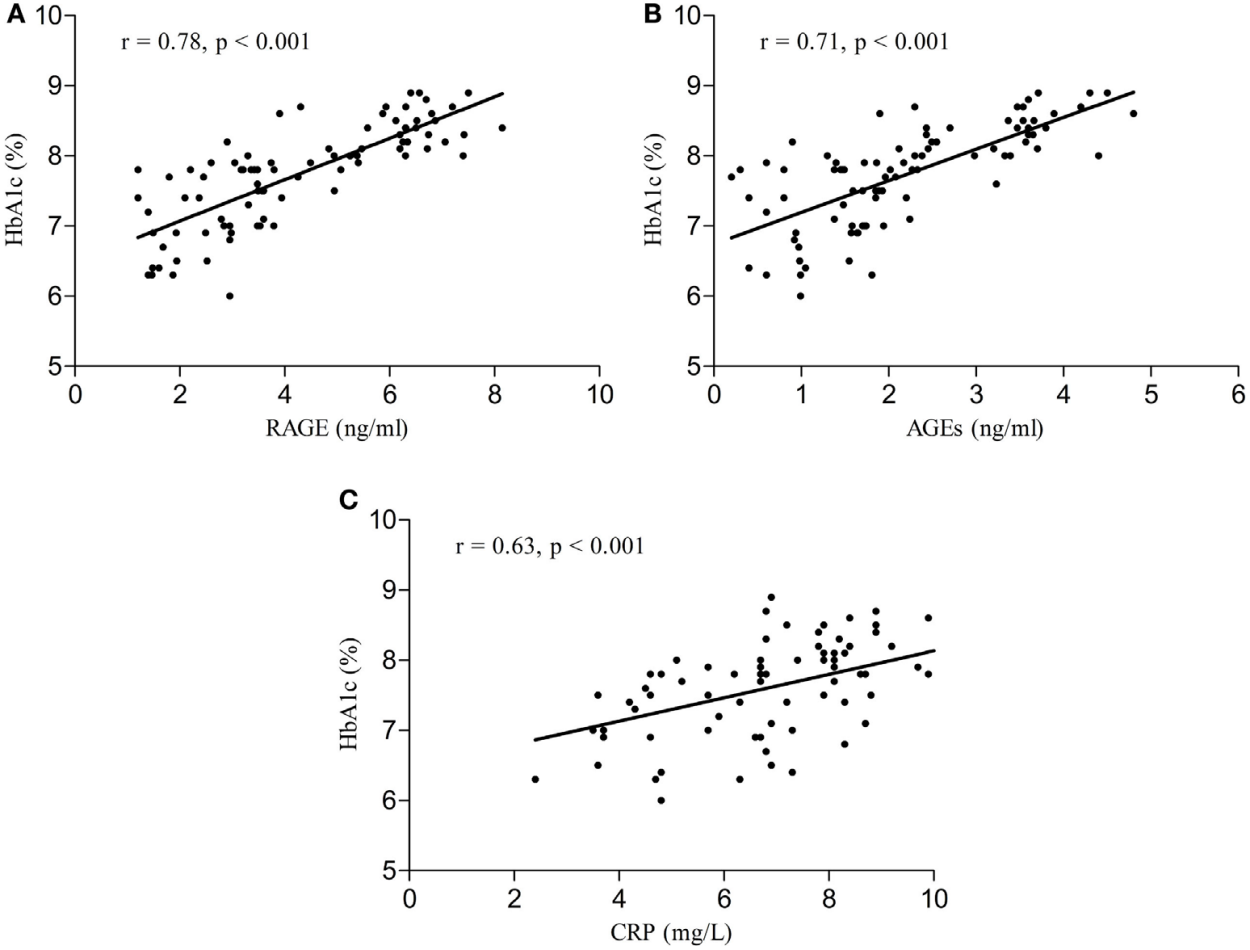
**(A)** Correlation of HbA1c with RAGE in group of diabetic elderly patients with MCI. **(B)** Correlation of HbA1c with AGEs in group of diabetic elderly patients with MCI. **(C)** Correlation of HbA1c with CRP in group of diabetic elderly patients with MCI.

**Figure 3:**
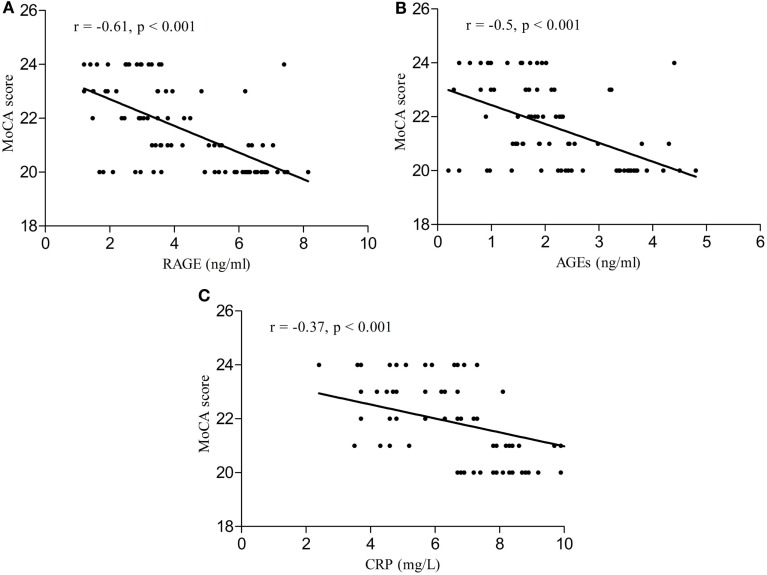
**(A)** Correlation of MoCA score with RAGE in group of diabetic elderly patients with MCI. **(B)** Correlation of MoCA score with AGEs in group of diabetic elderly patients with MCI. **(C)** Correlation of MoCA score with CRP in group of diabetic elderly patients with MCI.

Serum RAGE and AGEs levels were significantly increased in MCI patients compared to controls (*p* < 0.001). As expected, in group of diabetic elderly patients with MCI serum RAGE level was positively correlated with AGEs level (*r* = 0.85, *p* < 0.001) and with CRP level (*r* = 0.54, *p* < 0.001) (Figures 1A–C). Furthermore, RAGE, AGEs, and CRP concentrations were highly correlated with HbA1c levels (Figures 2A–C). We found also positive but weak correlation between these parameters and triglycerides levels and negative correlation with HDL levels. The results indicated that MoCA score was negatively correlated with RAGE, AGEs, and CRP levels (Figures 3A–C). Data are presented in Table 3.

**Table 3:** **Relationship of serum levels of AGEs, RAGE, and CRP with other clinical indicators in group of diabetic elderly patients with MCI**.

	AGEs	*p*	RAGE	*p*	CRP	*p*
	*r*		*r*		*r*	
MoCA score	−0.5	*p* < 0.001	−0.61	*p* < 0.001	−0.37	*p* < 0.001
HbA1c (%)	0.71	*p* < 0.001	0.78	*p* < 0.001	0.63	*p* < 0.001
Serum cholesterol (mmol/l)	0.1	0.33	0.12	0.25	0.35	0.001
Serum LDL-C (mmol/l)	0.06	0.6	0.08	0.43	0.27	0.01
Serum triglycerides (mmol/l)	0.26	0.014	0.37	*p* < 0.001	0.28	0.007
Serum HDL-C (mmol/l)	−0.2	0.056	−0.33	0.002	−0.19	0.06
AGEs (ng/ml)	1		0.85	*p* < 0.001	0.51	*p* < 0.001
RAGE (ng/ml)			1		0.54	*p* < 0.001
CRP (mg/l)					1	

*Significance, p < 0.05; r, correlation coefficient.

*BMI, body mass index; CHOL, total cholesterol; CRP, C-reactive protein; AGEs, advanced glycation end products; RAGE, receptor for advanced glycation end products; HbA1c, glycosylated hemoglobin; HDL-C, high-density lipoprotein cholesterol; LDL-C, low-density lipoprotein cholesterol; MoCA, Montreal Cognitive Assessment*.

### Logistic Regression Models

Because many factors can influence the results we constructed the univariate logistic regression models and finally multivariable regression model to determine the predictors of having MCI in elderly patients with type 2 diabetes. The independent variables entered in the model at step one were demographic variables (age, gender, education), duration of diabetes, glycemic control (HbA1c level), CVDs (MI, angina, stroke), cardiovascular risk factors (BMI, smoking status, hyperlipidemia, previous HA or use of HA drugs), microvascular complications, presence of depressive syndrome, number of co-morbid conditions, levels of total, LDL, HDL cholesterol, triglycerides, AGEs, RAGE, and CRP. The univariate logistic regression models revealed that variables which increased the likelihood of having been diagnosed with MCI in elderly patients with type 2 diabetes were higher levels of HbA1c, RAGE, AGEs, CRP, TG, lower level of HDL cholesterol, previous CVD, HA or use of HA drugs, hyperlipidemia, retinopathy, nephropathy, increased number of co-morbidities, older age, and less years of formal education (Table 4).

**Table 4:** **Assessment results of the risk of having MCI in a simple logistic regression model in elderly patients with type 2 diabetes**.

Variables analyzed	**β**	SE of **β**	*p*-value	OR	95% CI
Age (years)*	0.137	0.03	*p* < 0.001	1.15	1.08–1.22
Gender: female	0.2	0.1	0.12	1.2	0.49–1.59
Education (years)*	−0.639	0.09	*p* < 0.001	0.53	0.44–0.63
Smoked tobacco regularly	0.12	0.1	0.36	0.8	0.6–1.16
Duration of T2DM (years)*	0.097	0.02	*p* < 0.001	1.1	1.05–1.15
Previous stroke	0.41	0.27	0.14	1.5	0.87–2.58
Previous CVD*	1.43	0.16	*p* < 0.001	4.19	3.03–5.81
Previous HA or use of HA drugs*	0.88	0.22	*p* < 0.001	2.41	1.55–3.76
Hyperlipidemia*	0.72	0.21	0.001	2.01	1.35–3.12
Retinopathy*	0.8	0.14	*p* < 0.001	2.24	1.7–2.96
Nephropathy*	0.44	0.13	0.001	1.56	1.2–2.03
Neuropathy	0.11	0.01	0.45	1.12	0.82–1.53
Co-morbidity (*n*)*	0.426	0.05	*p* < 0.001	1.53	1.37–1.71
Depressive syndrome	0.03	0.01	0.8	0.96	0.73–1.27
BMI (kg/m^2^)	0.058	0.03	0.1	1.06	0.98–1.13
HbA1c (%)*	1.69	0.23	*p* < 0.001	5.47	3.45–8.67
Serum cholesterol (mmol/l)	0.01	0.003	0.95	1.01	0.99–1.01
Serum LDL-C (mmol/l)	0.01	0.004	0.74	1.01	0.99–1.01
Serum triglycerides (mmol/l)*	0.02	0.004	*p* < 0.001	1.02	1.01–1.02
Serum HDL-C (mmol/l)*	−0.09	0.018	*p* < 0.001	0.91	0.87–0.94
CRP (mg/L)*	0.64	0.08	*p* < 0.001	1.9	1.62–2.22
AGEs (ng/ml)*	1.15	0.16	*p* < 0.001	3.17	2.31–4.34
RAGE (ng/ml)*	1.19	0.16	*p* < 0.001	3.28	2.42–4.45

*Significance, *p* < 0.05.

*β, regression coefficient; CI, confidence interval for odds ratio; OR, odds ratio; SE, standard error; T2DM, diabetes type 2; CVD, cardiovascular disease; HA, hypertension; BMI, body mass index; CHOL, total cholesterol; CRP, C-reactive protein; AGEs, advanced glycation end products; RAGE, receptor for advanced glycation end products; HbA1c, glycosylated hemoglobin; HDL-C, high-density lipoprotein cholesterol; LDL-C, low-density lipoprotein cholesterol*.

Table 5 shows the results of modeling the risk of having MCI by multivariable regression. All variables presented in Table 3 were introduced to this model. The multivariable model was optimized by the stepwise approach. HA or use of HA drugs, previous CVD, higher level of RAGE and CRP, older age and less years of formal education are the factors increasing the likelihood of having MCI in elderly patients with type 2 diabetes.

**Table 5:** **Assessment results of the risk of having MCI in a multivariable logistic regression model in elderly patients with type 2 diabetes**.

Variables analyzed	**β**	SE of **β**	*p*-value	OR	95% CI
Age (years)*	0.11	0.05	0.039	1.11	1.0–1.23
Education (years)*	−0.45	0.12	*p* < 0.001	0.64	0.5–0.82
Previous CVD*	1.2	0.24	*p* < 0.001	3.34	2.06–5.41
Previous HA or use of HA drugs*	1.24	0.41	0.002	3.48	1.56–7.76
CRP (mg/l)*	0.44	0.13	0.001	1.55	1.21–1.99
RAGE (ng/ml)*	0.67	0.23	0.004	1.95	1.24–3.06

*Significance, *p* < 0.05.

*β, regression coefficient; CI, confidence interval for odds ratio; OR, odds ratio; SE, standard error; CVD, cardiovascular disease; HA, hypertension; CRP, C-reactive protein; RAGE, receptor for advanced glycation end products*.

## Discussion

Our results show that patients with MCI had significantly higher CRP levels than controls. The increased CRP level is a predictor of cognitive impairment in elderly patients with type 2 diabetes. This result is consistent with recent reports. CRP – an acute-phase protein – is commonly used marker, which participates in low-grade inflammation in diabetes. It is mainly associated with the risk of DM complications. Other researchers had found serum levels of CRP were associated with cognition impairment and inversely correlated with MMSE and MoCA scores (Ge et al., 2013). In other studies, low-grade inflammation as reflected by serum CRP concentrations was found in subjects with cognitive dysfunction without dementia and in patients with non-amnestic MCI (Haan et al., 2008; Roberts et al., 2009). CRP is also associated with increased risk of CVD (Ridker et al., 1998). In our study, CRP as well as CVD is independent predictors of MCI in diabetic patients. Thus, the mechanism of the involvement of low-grade inflammation in cognitive impairment is not explained entirely by the presence of CVD in diabetic patients. CRP could initiate immune cascade reactions leading to neurodegeneration via activating the complement system (Eagan et al., 2012).

Like CRP, AGEs–RAGE system stimulated by hyperglycemia could be responsible for the deterioration of cognition through an immune inflammatory pathway. AGEs – the products of non-enzymatic glycation on lysine and arginine residues accumulate in diabetic tissues – plays the important role in pathogenesis of diabetic complications. AGEs binds their receptor – RAGE and modulates cellular properties. Stimulation of RAGE results in activation of NADPH oxidase and generation of oxidation stress. Reactive oxygen species activate also formation of AGEs. In that way, the vicious circle is created – hyperglycemia induce AGEs generation, then after binding to RAGE increase oxidation stress and further production of AGEs (Kislinger et al., 1999; Wautier et al., 2001). Expression of RAGE is increased in CVD and in diabetes.

The frequency of cardiac events is higher and the progression of atherosclerosis is bigger in patients with diabetes compared to age-matched healthy controls. A lot of studies showed elevation of RAGE in diabetes and its association with diabetic complications and severity of the disease (Matsunaga-Irie et al., 2004; Yan et al., 2009). There are also data about increasing levels of AGEs with age and it is further enhancement by hyperglycemic state (Ulrich and Cerami, 2001). The interaction between AGEs and RAGE could result in higher inflammation in vascular wall via stimulation of oxidative stress. Activation of endothelial, smooth muscle cells and mononuclear phagocytes is highly associated with diabetic complications, such as nephropathy, retinopathy, and atherosclerosis (Schmidt et al., 2000b; Win et al., 2012).

Stimulation of RAGE play crucial role in the pathogenesis of T2DM and its complications, but RAGE is also an important cell-signaling receptor involved cognitive impairment. RAGE expression was found in different structures of the brain, in microglia, and astrocytes. In Alzheimer disease, RAGE expression was increased in inferior frontal cortex and hippocampus. The stimulation of RAGE in neurons can lead to increased inflammation through activation of the transcription faction NF-κB. In diabetes neuroinflammation, vascular injury and also activation of microglia could be driven by RAGE and the cascade of chemokines and cytokines (Lue et al., 2012).

In our study, AGEs and RAGE levels were significantly elevated in MCI subjects. The results are consistent with one study, which reported also higher level of AGEs in MCI subject with T2DM (Chen et al., 2011). The authors proposed possible the mechanism by which AGEs induce MCI in patients with type 2 diabetes. It is possible that AGEs stimulate endothelial dysfunction and followed the increased permeability of blood vessels. Thus, AGEs can accumulates in vascular wall and cause its injury. Other explanation could be higher inflammation and neural damage via inducing oxidative stress mediated by AGEs–RAGE interactions. AGEs could also activate microglia and destroy microtubular structure resulting in dysfunction of neurons (Chen et al., 2011).

In opposite to this result recent study was evaluated whether plasma levels of RAGE are altered in MCI and Alzheimer’s disease. The authors did not found higher levels of RAGE in MCI but the study was performed among only 24 patients without vascular disease (only 16% of them had diabetes) (Marksteiner et al., 2014).

Expectedly, we found significant relationships between AGEs, RAGE, CRP, and glycosylated hemoglobin. Subjects with MCI had also more micro complications. Sustained hyperglycemia and development of diabetes complications can induce chronic low-grade inflammation and cell activation via AGEs–RAGE interaction. In agreement with our results, other studies reported that persistent hyperglycemia, indicated by elevated HbA1c, is an independent risk factor for the cognitive dysfunction (Ryan and Geckle, 2000).

In our previous work, we found that the presence of depressive syndrome is associated with higher levels of inflammatory markers in elderly patients with diabetes (Gorska-Ciebiada et al., 2015). Therefore, we put this parameter into logistic regression models; however, we revealed that the presence of depressive syndrome have no influence on the results.

### Limitations

This study provides important insights into AGEs–RAGE system disturbances and low-grade inflammation pathologies underlying cognitive impairment in older diabetic patients; however, it is not without limitations. First, because our study population was relatively small, our results should be interpreted with caution. Second, the study was not designed as longitudinal prospective investigation. There have been few studies in which the cognitive declines in elderly diabetics were prospectively observed. However, the precise mechanisms underlying T2DM-related cognitive dysfunction or the development of dementia have not yet been elucidated. Furthermore, this investigation was limited to patients with diabetes, and therefore, an association between AGEs, RAGE, and CRP with other parameters in subjects without diabetes should also be assessed.

## Conclusion

In summary, serum AGEs, RAGE, and CRP are increased in the circulation of MCI elderly diabetic patients compared to controls. Higher level of RAGE and CRP, HA or use of HA drugs, previous CVD, older age and less years of formal education are the factors increasing the likelihood of having MCI in elderly patients with type 2 diabetes. The precise mechanisms responsible for this finding are not entirely clear. A larger population-based prospective study needs to be performed to further confirm the relationship between AGEs, RAGE, and the cognitive decline or progress to dementia. As an option, targeting the AGEs–RAGE system in diabetes especially with cognitive impairments through specific pharmacologic interventions might result in a clinical benefit for these patients.

## Conflict of Interest Statement

The authors declare that the research was conducted in the absence of any commercial or financial relationships that could be construed as a potential conflict of interest.
